# Absorb® bioresorbable scaffold in “established” versus “off-label” coronary lesions: 5-year data from the GABI-R® registry

**DOI:** 10.1007/s00392-025-02707-3

**Published:** 2025-06-30

**Authors:** Aydin Huseynov, Michael Behnes, Holger Nef, Thomas Riemer, Steffen Schneider, Thomas Pfannebecker, Stephan Achenbach, Julinda Mehilli, Thomas Münzel, Tommaso Gori, Jochen Wöhrle, Ralf Zahn, Johannes Kastner, Axel Schmermund, Gert Richardt, Christian W. Hamm, Ibrahim Akin

**Affiliations:** 1 First Department of Medicine-Cardiology, Faculty of Medicine Mannheim, University Medical Centre Mannheim, University of Heidelberg, Theodor-Kutzer-Ufer 1-3, 68167 Mannheim, Germany; 2 DZHK (German Centre for Cardiovascular Research), Partner Site Heidelberg/Mannheim, Mannheim, Germany; 3 Heart Centre Segebereger Kliniken GmbH, Bad Segeberg, Germany; 4 Department of Cardiology, University of Giessen, Giessen, Germany; 5 IHF GmbH, Institut Für Herzinfarktforschung, Ludwigshafen, Germany; 6 Abbott Vascular Deutschland, Wetzlar, Germany; 7 Department of Cardiology, Friedrich-Alexander University Erlangen-Nürnberg, Erlangen, Germany; 8 Medical Clinic I, Hospital Landshut-Achdorf, Landshut, Germany; 9 University Medical Center, Johannes Gutenberg University Mainz, Mainz, Germany; 10 Department of Cardiology, Clinic Friedrichshafen, Friedrichshafen, Germany; 11 Department of Cardiology, Heart Centre Ludwigshafen, Ludwigshafen, Germany; 12 Department of Cardiology, University of Vienna Medical School, Vienna, Austria; 13 Bethanien Hospital, Frankfurt, Germany; 14 Department of Cardiology, Asklepios Klinik Bad Oldesloe, Bad Oldesloe, Germany

**Keywords:** Bioresorbable scaffold, Coronary artery disease, Off-label use, 5-year follow-up

## Abstract

**Background:**

The potential benefits of bioabsorbable stents can be better assessed over the long term. The implantation of bioresorbable scaffold (BRS) in situations with off-label indications provides real-world insights into how clinical events differ in contrast to standard proved indications.

**Objectives:**

The study provides long-term follow-up data about the use of bioresorbable scaffold (BRS) for off-label compared with approved indications.

**Methods:**

Five-year outcome data of an everolimus-eluting, poly-L-lactic acid–based bioresorbable scaffold system (ABSORB, Abbott Vascular, Santa Clara, CA, USA) were evaluated in the prospective, non-interventional, multicenter real-world German-Austrian ABSORB-RegIstRy (GABI-R). The patients were enrolled from a total of 93 centers. Data processing and prospective follow-up were conducted centrally and independently of industry.

**Results:**

A total of 3082 patients were enrolled between 2013 and 2016. Most patients were included into the off-label group (2317, 75.2%). ST-elevation myocardial infarction (STEMI) was significantly more common in the off-label group (35.9% vs. 27.8%, *p* = 0.003), and the extent of coronary heart disease was higher in the off-label group (coronary 3 vessel disease 28.4% vs. 22.4%, *p* < 0.001). Patients with off-label indications had statistically significant higher rates of stent thrombosis after 30 days (1.08% vs. 0.26%, *p* = 0.04) and target vessel failure (TVF) after 6 months (4.62% vs. 2.61%, *p* = 0.02).

**Conclusions:**

The off-label use of BRS is associated with a higher rate of stent thrombosis in the short term and in the long term with higher MACE events considering more complex lesions and a higher morbidity. In the long term, there are no differences regarding stent thrombosis.

**Graphical Abstract:**

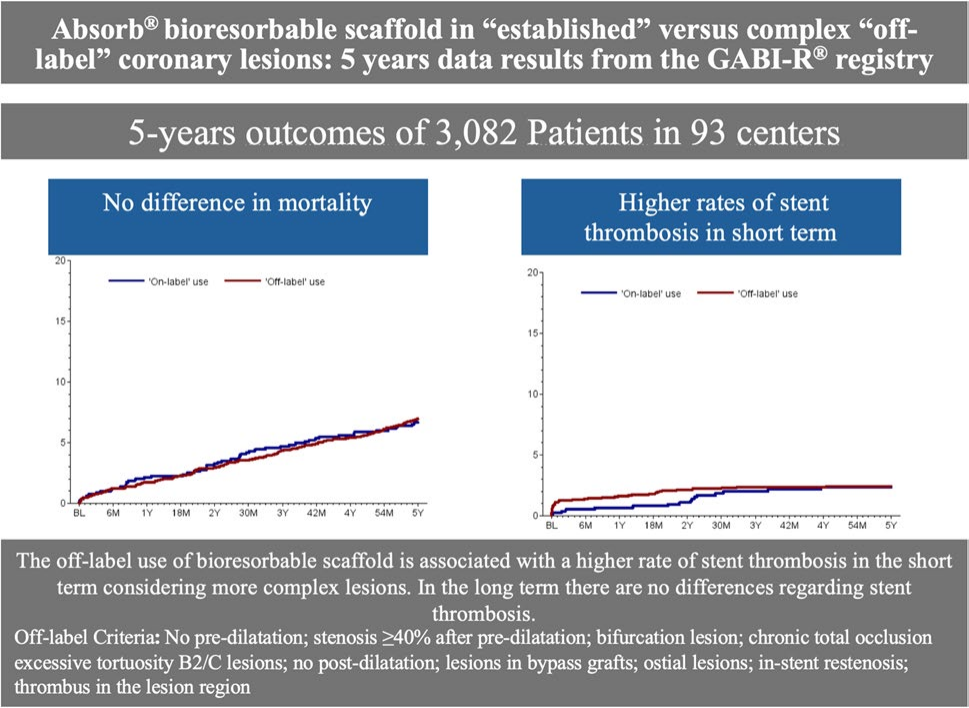

## Introduction

Bioabsorbable scaffolds (BRS) were a novel approach that could provide transient vessel support with drug delivery capability without the long-term limitations of the metallic drug-eluting stents (DES), such as persistent inflammatory reaction with abnormal structure and function of the endothelium around the stent struts [1–3]. The first use of BRS was in patients with very simple de novo type A coronary stenoses [4, 5]. In contrast to the selected study patients, the average patients often have significantly complex lesions that are treated interventionally. Even if this is common practice, these indications that were not primarily treated in the approval studies are considered as off-label indications. Older studies with coronary stents have shown that the complication rate is significantly higher with off-label indications, which is due to more complex lesion morphology and comorbidities [6–8]. Use of BRS for more complex lesions was associated with a higher rate of clinical endpoints considering more complex lesions and higher morbidity in short term [9].

The long-term results after treatment with a BRS are of particular interest, as the advantages of absorption are expected to become apparent after complete absorption. Real-world multicenter German-Austrian ABSORB RegIstRy (GABI-R) provides 5-year follow-up data about the use of BRS in the off-label and approved indications.

## Methods

The GABI-R multicenter, prospective, observational registry was established to provide comprehensive data on the acute and long-term safety and therapeutic outcomes of patients with coronary artery disease (CAD) treated with ABSORB (Abbott Vascular, Santa Clara, California, USA) BRS. The follow-up period was at least 5 years. The details of the study design have been described in detail previously [10]. Between November 2013 and January 2016, a total of 3264 patients in 93 centers in Germany and Austria were included in the registry. This was an observational study in which a patient’s participation had no impact on their treatment. The final decision as to whether percutaneous coronary angioplasty (PCI) was performed with an ABSORB BRS rested with the treating physician. The study was conducted in accordance with the principles of the Declaration of Helsinki and the International Conference on Harmonization of Good Clinical Practice. The ethics committees of all participating centers approved the registry protocol. The study was registered with Clinicaltrials.gov (NCT 02066623), and all patients provided written informed consent.

## Procedure

Given the real-world design, the specific protocols for lesion pre-dilatation during implantation, as well as BRS post-dilatation, were not predetermined and were instead at the discretion of the operator. Similarly, the methods for sizing BRS or balloon were also left to the operator’s judgment. Dual antiplatelet therapy, comprising aspirin along with either clopidogrel, prasugrel, or ticagrelor, was recommended for a minimum duration of 12 months. Standard loading and maintenance dosages as indicated on the label were employed.

### Study groups and clinical outcomes

We conducted a comparison between patients who received BRS for off-label indications and those treated with BRS for on-label use. Off-label BRS utilization, as outlined in the study protocol, was characterized if at least one of the following criteria was met: (1) absence of pre-dilatation; (2) presence of stenosis ≥ 40% after pre-dilatation; (3) presence of a bifurcation lesion; (4) chronic total occlusion (CTO); (5) excessive tortuosity of the lesion; (6) lesions categorized as B2/C; (7) absence of post-dilatation; (8) lesions in bypass grafts; (9) lesions in ostia; (10) in-stent restenosis (ISR); (11) presence of thrombus in the lesion region. Patients with vessel sizes < 2.25 mm or > 4.00 mm were excluded from the study due to contraindications for Absorb BRS in these cases. We defined stent thrombosis and target lesion failure (TLR) as the primary outcomes of the study. The Academic Research Consortium (ARC) definitions were employed to identify probable and definite instances of stent thrombosis [11]. All reported occurrences of stent thrombosis were reviewed and approved by the study’s Critical Event Committee. Additional clinical outcomes, such as myocardial infarction (MI), all-cause mortality, and major adverse cardiovascular events (MACE)—encompassing stent thrombosis, death, or MI—were utilized as secondary endpoints in the study.

### Data collection

Data were gathered using an electronic Case Report Form administered by the “Institut für Herzinfarktforschung GmbH” (IHF). The patients received written notifications by mail, prompting them to complete “follow-up” and “quality of life” questionnaires and return them to IHF. All datasets were analyzed in a blinded fashion, independent of any sponsorship. In cases of non-response, participants were contacted via telephone or the registry’s registration was utilized to gather follow-up data. IHF conducted random spot-checks, overseen by a monitor, to compare authentic data with the documented entries in the registry’s database.

### Statistical analysis

Baseline characteristics were presented as mean ± standard deviation (SD) for continuous variables and as percentages for categorical variables. Clinical characteristics were compared using either the chi-squared test or the Mann–Whitney–Wilcoxon test. Odds ratios and 95% confidence intervals were provided for outcome frequencies whenever possible. Statistical significance was defined as a two-tailed *p*-value less than 0.05. Missing values were imputed using modal values for binary variables and median values for metric variables. Kaplan–Meier estimates were employed to analyze the study endpoints. Statistical analyses were conducted using the SAS software, version 9.4 (SAS Institute Inc., Cary, North Carolina, USA).

## Results

In total, data from 3082 patients with implanted BRS were analyzed. If at least one off-label criteria was met, the patient was designated as a patient from the off-label group. The majority of patients were in the off-label group (2317, 75.2%), and approximately one in four patients was in the on-label group (765, 24.8%).

### Baseline and periprocedural characteristics

The relevant baseline characteristics, such as previous illnesses, cardiovascular risk factors, type of presentation, and the extent of coronary heart disease, are summarized in Table 1. The relevant demographic characteristics such as age, gender, and cardiovascular risk factors, except for smoking (56.4% vs. 61.9%, *p* = 0.01), are represented without any differences between both groups. Regarding the preprocedural medications, ACE inhibitors (33.4% vs. 28.5%, *p* = 0.01) and ADP antagonists (20.8% vs. 16.1%, *p* = 0.005) were more common in the off-label group. Significant differences were noted in the type of clinical presentation; ST-elevation myocardial infarction (STEMI) was significantly more common as a reason for admission in the off-label group (35.9% vs. 27.8%, *p* = 0.003), and as a result, flow characteristics were worse in the off-label group (TIMI 0 flow 15.8% vs. 6.0%, *p* < 0.001; TIMI 3 flow 16.6% vs. 14.3%, *p* < 0.001). Furthermore, the extent of coronary heart disease was higher in the off-label group (coronary 3 vessel disease 28.4% vs. 22.4%, *p* < 0.001). Lesion characteristics before BRS implantation are listed in Table 2. RCX stenoses were described more frequently in off-label patients (44.8% vs. 38.6%, *p* = 0.003), while RCA interventions were less common (66.0% vs. 75.7%, *p* < 0.001).

**Table 1:** Clinical characteristics

	Total	Off-label	On-label	*p*-value*	OR (95% -CI)
Patients	3082	2317	765	n.d	-
Age, years	60.86 ± 10.98	60.89 ± 11.00	60.78 ± 10.93	0.86	-
Female gender	23.0%	23.1%	22.5%	0.71	-
Hypertension	73.4%	74.4%	70.5%	0.04	1.21 (1.01–1.45)
Diabetes mellitus	20.8%	20.6%	21.3%	0.67	0.96 (0.78–1.17)
Previous MI	22.0%	22.0%	22.1%	0.92	0.99 (0.81–1.21)
Previous CABG	2.5%	2.6%	2.1%	0.40	1.27 (0.72–2.21)
Hyperlipoproteinemia	56.3%	55.9%	57.3%	0.53	0.95 (0.80–1.12)
Family history of CAD	41.0%	41.4%	39.9%	0.49	1.06 (0.89–1.27)
Atrial fibrillation	6.8%	7.0%	6.1%	0.39	1.16 (0.83–1.63)
Current or previous smoker	57.8%	56.4%	61.9%	0.01	0.80 (0.67–0.95)
GFR, ml/Min	79.39 ± 23.59	79.53 ± 23.52	78.91 ± 23.87	0.87	-
Preprocedural medical treatment					
ASA	47.9%	48.4%	46.5%	0.36	1.08 (0.92–1.27)
ADP antagonist	19.6%	20.8%	16.1%	0.005	1.37 (1.10–1.70)
Oral anticoagulants	5.0%	5.1%	4.7%	0.68	1.09 (0.74–1.59)
Statins	42.3%	42.8%	40.7%	0.32	1.09 (0.92–1.29)
ß-Blockers	43%	43.7%	41.0%	0.18	1.12 (0.95–1.32)
ACE inhibitors AT1 Blocker/ARB	32.2% 17.0%	33.4% 16.5%	28.5% 18.3%	0.01 0.25	1.25 (1.05–1.50) 0.88 (0.71–1.09)
Clinical presentation					
Acute coronary syndrome	51.6% (1589/3081)	51.5% (1193/2317)	51.8% (396/764)	0.87	0.99 (0.84–1.16)
Unstable AP	22.6% (359/1589)	21.0% (250/1193)	27.5% (109/396)	0.007	0.70
NSTEMI	43.5% (692/1589)	43.2% (515/1193)	44.7% (177/396)	0.60	0.94 (0.75–1.18)
STEMI	33.9% (538/1589)	35.9% (428/1193)	27.8% (110/396)	0.003	1.45 (1.13–1.87)
Number of vessel disease					
1	42.0%	41.1%	44.6%	0.09	0.87 (0.74–1.02)
2	31.1%	30.5%	33.1%	0.18	0.89 (0.74–1.06)
3	26.9%	28.4%	22.4%	< 0.001	1.38 (1.14–1.67)

Values are mean ± standard deviation (SD) or number and percentage (*n*, %)

*ACE* angiotensin-converting-enzyme, *AP* angina pectoris, *ARB* angiotensin receptor blocker, *AT1* angiotensin1, *BMI* body mass index, *CABG* coronary artery bypass graft, *CAD* coronary artery disease, *GFR* glomerular filtration rate, *NSTEMI* non-ST-elevation myocardial infarction, *MI* myocardial infarction, *STEMI* ST-elevation myocardial infarction

^*^Comparison between off-label and on-label use. The *p* values are from chi-squared test or Mann–Whitney–Wilcoxon test

**Table 2:** Lesion characteristics

	Total	Off-label	On-label	*p*-value*	OR (95% CI)
Target vessel					
Stenosis ≥ 50% in left main	1.9% (58)	1.9% (44)	1.8% (14)	0.90	1.04 (0.57–1.91)
Intervention in left main	39.7% (23/58)	36.4% (16/44)	50.0% (7/14)	0.36	0.57 (0.17–1.92)
Stenosis ≥ 50% in left anterior descending	67.6% (2083)	68.4% (1585)	65.1% (498)	0.09	1.16 (0.98–1.38)
Intervention in left anterior descending	74.7% (1557/2083)	74.8% (1186/1585)	74.5% (371/498)	0.88	1.02 (0.81–1.28)
Stenosis ≥ 50% in left circumflex	43.3% (1333)	44.8% (1038)	38.6% (295)	0.003	1.29 (1.09–1.53)
Intervention in left circumflex	59.5% (793/1333)	59.3% (616/1038)	60.2% (177/295)	0.79	0.96 (0.74–1.26)
Stenosis ≥ 50% in right coronary artery	48.6% (1499)	48.6% (1125)	48.9% (374)	0.87	0.99 (0.84–1.16)
Intervention in right coronary artery	68.4% (1026/1499)	66.0% (743/1125)	75.7% (283/374)	< 0.001	0.63 (0.48–0.82)
Stenosis ≥ 50% in CABG	0.7% (21)	0.8% (19)	0.3% (2)	0.10	3.15 (0.73–13.57)
Intervention in CABG	28.6% (6/21)	31.6% (6/19)	0.0%	0.35	-
Lesion morphology					
A/B1	64.4%	54.0%	100%	< 0.001	-
B2/C	35.6%	46.0%	0.0%	< 0.001	-
TIMI-flow pre-PCI					
TIMI 0	13.6%	15.8%	6.0%	< 0.001	2.97 (1.85–4.83)
TIMI 1	7.7%	7.5%	8.6%	0.28	0.86 (0.65–1.13)
TIMI 2	16.1%	16.6%	14.3%	0.11	1.19 (0.96–1.48)
TIMI 3	62.6%	60.1%	71.1%	< 0.001	0.61 (0.52–0.72)
Lesion characteristics					
De novo vessel	94.7%	93.6%	98.4%	< 0.001	0.23 (0.13–0.41)
In-stent Re-stenosis	0.8%	1.0%	0.0%	0.004	-
Treated bifurcation	2.1%	2.7%	0.0%	< 0.001	-
Ostial lesion	0.8%	1.0%	0.0%	0.003	-
Vessel diameter, mm	3.09 ± 0.42	3.08 ± 0.39	3.14 ± 0.76	0.51	-
Severe calcification	3.4%	4.1%	0.8%	< 0.001	4.98 (2.31–10.73)
Severe tortuosity	1.0%	1.2%	0.0%	0.001	-
Stenosis before PCI, %	86.41	86.83	85.01	< 0.001	-
Lesion length > 34 mm (at least one)	5.6%	6.5%	2.3%	< 0.001	2.91 (1.86–4.55)
Complete occlusion	5.7% (207)	6.5% (183)	2.9% (24)	< 0.001	2.32 (1.51–3.58)
Acute (< 24 h)	49.3% (102/207)	45.9% (84/183)	75% (18/24)	< 0.007	0.28 (0.11–0.75)
Subacute (> 24 h)	14% (29/207)	12.6% (23/183)	25% (6/24)	0.10	0.43 (0.16–1.20)
Chronic	36.7% (76/207)	41.5% (76/183)	0	< 0.001	-

Values are mean ± standard deviation (SD) or number and percentage (*n*, %)

*ACC/AHA* American College of Cardiology/American Heart Association, *CABG* coronary artery bypass graft, *PCI* percutaneous coronary intervention, *TIMI* thrombosis in myocardial infarction study group grade flow

^*^Comparison between off-label and on-label use. The *p*-values are from chi-squared test or Mann–Whitney–Wilcoxon test

Relevant intervention characteristics are shown in Table 3. Significant differences can be seen in access site (46.3% vs. 52.6%, *p* = 0.002), predilatation characteristics, and average BRS (23.0 vs. 18.0 mm, *p* < 0.001). The relevant features such as post-dilatation (69.9% vs. 100%, *p* < 0.001) and application of PSP criteria (pre-dilatation, sizing, and post-dilatation) (5.1% vs. 11.6%, *p* < 0.001) were more common in the on-label group. Table 4 contains data on relevant periprocedural complications and discharge characteristics. Overall, complications occurred very rarely and there were no relevant differences between the groups.

**Table 3:** Procedural characteristics

	Total	Off-label	On-label	*p*-value*	OR (95% CI)
Procedure duration, Min	58.95 ± 28.91	59.39 ± 29.47	57.60 ± 27.13	0.35	-
Area dose product, cGy * cm^2^	4804.96 ± 4698.65	4875 ± 4824.99	4592.37 ± 4288.81	0.25	-
Radiation time, min	11.86 ± 8.23	12.08 ± 8.53	11.21 ± 7.23	0.16	-
Amount of contrast, mL	175.09 ± 74.52	177.37 ± 76.12	168.17 ± 69.04	0.01	-
Femoral access	52%	53.6%	47.4%	0.003	1.28 (1.09–1.51)
Radial access	47.8%	46.3%	52.6%	0.002	0.78 (0.66–0.91)
Patient had predilatation, s	95.6%	94.1%	100%	< 0.001	-
Maximum balloon diameter, mm	2.76 ± 0.47	2.71 ± 0.49	2.91 ± 0.39	< 0.001	-
Maximum balloon length, mm	15.98 ± 4.50	16.17 ± 4.57	15.38 ± 4.23	< 0.001	-
Maximum balloon pressure, bar	13.60 ± 3.29	13.69 ± 3.26	13.32 ± 3.37	< 0.001	-
Stenosis after pre-dilatation, %	29.30 ± 20.88	32.20 ± 22.22	22.70 ± 13.61	< 0.001	-
BRS implanted	100%	100%	100%	-	-
Only BRS	86.60%	86%	86.1%	0.93	0.99 (0.78–1.25)
Both BRS and stent(s) implanted	14.0%	14.0%	13.9%	0.93	1.01 (0.80–1.28)
Average diameter, mm	3.0	3.0	3.0	-	-
Total BRS length, mm	23.0	23.0	18.0	< 0.001	-
Maximum pressure, bar	13.45 ± 2.68	13.55 ± 2.67	13.11 ± 2.66	< 0.001	-
Post-PCI characteristics					
Residual stenosis after PCI, %	2.94 ± 9.49	3.07 ± 9.72	2.51 ± 8.68	0.10	-
Intravascular imaging after PCI (IVUS, OCT)	4.1%	4.2%	3.9%	0.69	1.09 (0.73–1.62)
MLD, mm	2.47 ± 0.92	2.40 ± 0.97	2.74 ± 0.60	0.03	-
MLA, mm^2^	5.28 ± 2.38	5.15 ± 2.42	5.88 ± 2.17	0.13	-
TIMI 0	0.5%	0.6%	0.1%	0.10	4.72 (0.62–35.64)
TIMI 1	0.2%	0.2%	0.0%	0.15	-
TIMI 2	1.1%	1.2%	0.6%	0.14	2.01 (0.78–5.15)
TIMI 3	98.3%	98.0%	99.3%	0.01	0.35 (0.15–0.82)
Patient had post-dilatation(s)	77.4%	69.9%	100%	< 0.001	-
Maximum balloon diameter, mm	3.26 ± 0.45	3.20 ± 0.46	3.41 ± 0.36	< 0.001	-
Maximum balloon length, mm	15.22 ± 4.23	15.50 ± 4.33	14.57 ± 3.91	< 0.001	-
Maximum balloon pressure, bar	16.72 ± 4.04	16.80 ± 4.11	16.53 ± 3.87	0.16	-
PSP implanted BRS	6.6%	5.1%	11.6%	< 0.001	0.41 (0.31–0.54)
Non-PSP implanted BRS	93.6%	94.9%	88.5%	< 0.001	2.50 (1.90–3.29)

Values are mean ± standard deviation (SD) or number and percentage (*n*, %)

*BRS* bioresorbable scaffold; *IVUS* intravascular ultrasound, *MLA* minimum lumen area, *MLD* minimum lumen diameter, *OCT* optical coherence tomography, *PCI* percutaneous coronary intervention, *PSP* pre-dilatation scaffold sizing and post-dilatation, *QCA* quantitative coronary angiography, *TIMI* thrombosis in myocardial infarction study group grade flow

^*^Comparison between off-label and on-label use. The *p* values are from Chi-squared test or Mann–Whitney–Wilcoxon test

**Table 4:** Discharge medication and complications

	Total	Off-label	On-label	*p*-value*	OR (95% CI)
Periprocedural events					
Death	0.0%	0.0%	0.0%	n.d	
MI	0.3%	0.3%	0.23%	0.72	1.32 (0.28–6.23)
Stroke	0.1%	0.1%	0.0%	0.42	-
Coronary thrombosis	0.4%	0.4%	0.3%	0.51	1.98 (0.24–16.48)
Medication at discharge					
Acetylsalicylic acid	97.3%	97.1%	97.9%	0.24	0.72 (0.41–1.25)
Clopidogrel	43.9%	43.9%	43.7%	0.87	0.95 (0.55–1.66)
Prasugrel	34.2%	34.4%	33.8%	0.78	1.03 (0.86–1.22)
Ticagrelor	21.9%	21.7%	22.5%	0.64	0.95 (0.78–1.16)
Oral anticoagulation	7.9%	8.3%	6.8%	0.19	1.24 (0.90–1.70)
Discharge status					
Alive at discharge	99.8%	99.8%	99.9%	0.64	0.61 (0.07–5.19)
Deceased before discharge	0.2%	0.2%	0.1%	0.64	1.65 (0.19–14.16)

Values are mean ± standard deviation (SD) or number and percentage (*n*, %)

*MI* myocardial infarction

^*^Comparison between off-label and on-label use. The *p* values are from chi-squared test or Mann–Whitney–Wilcoxon test

### Follow-up data

**Fig. 1:**
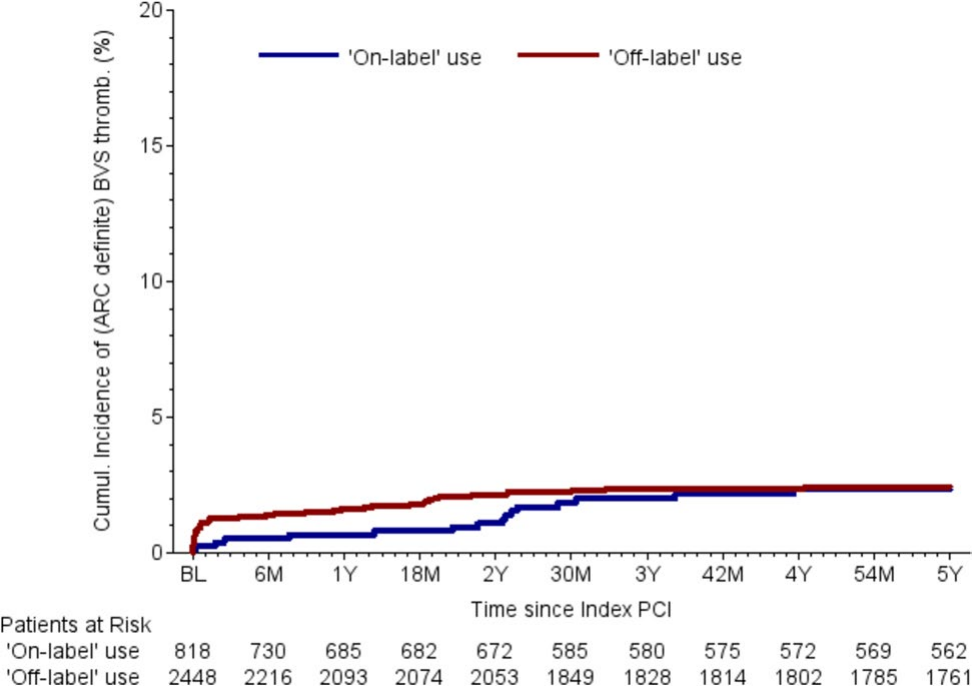
Incidence of BRS thrombosis

**Fig. 2:**
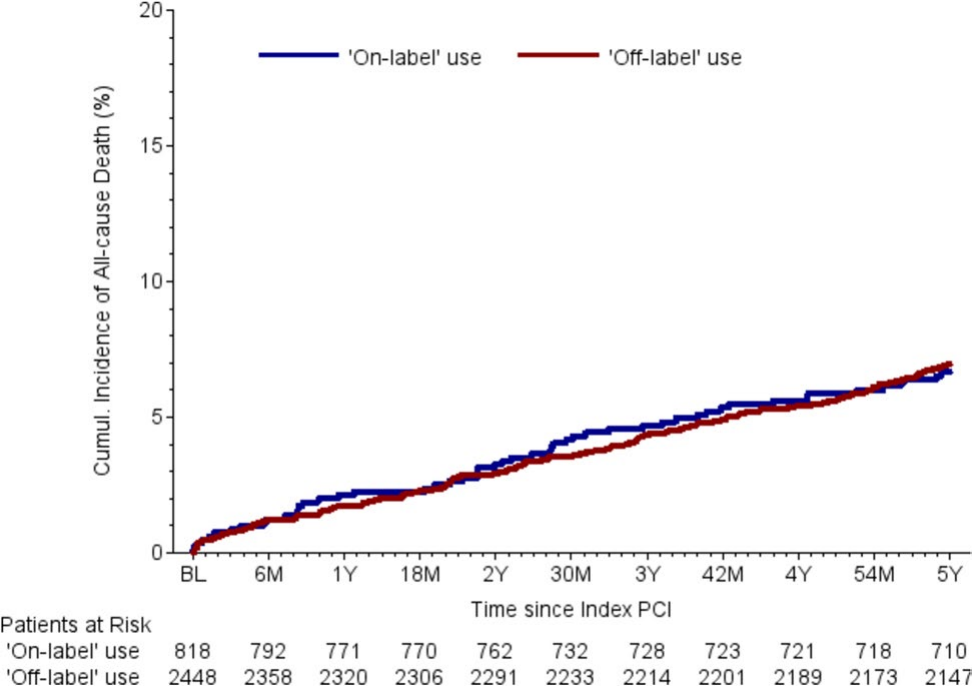
Cumulative incidence of all-cause mortality

**Fig. 3:**
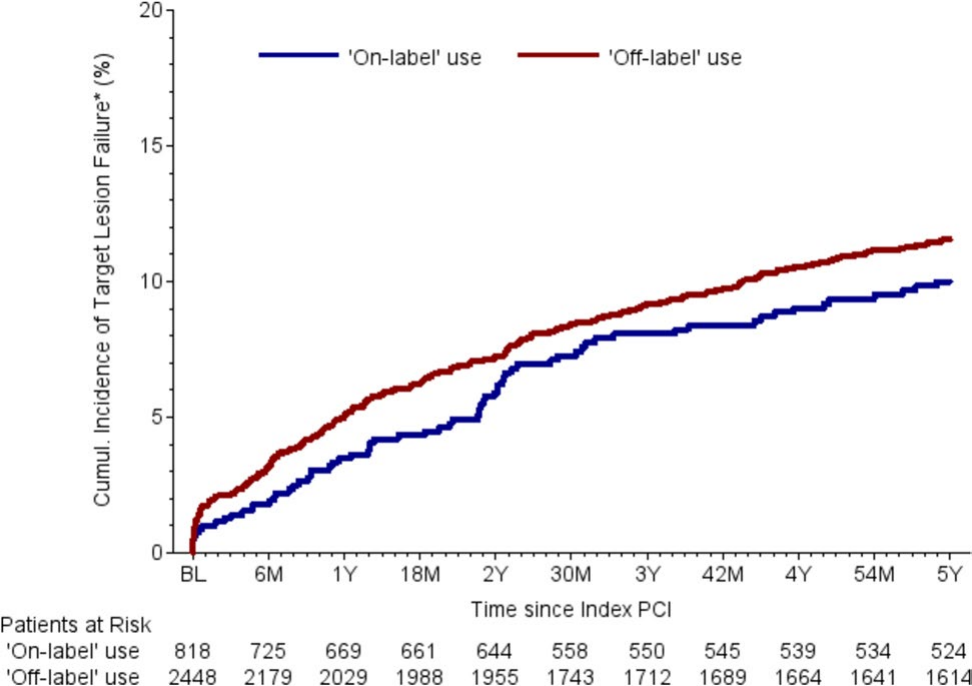
Incidence of target lesion failure

**Fig. 4:**
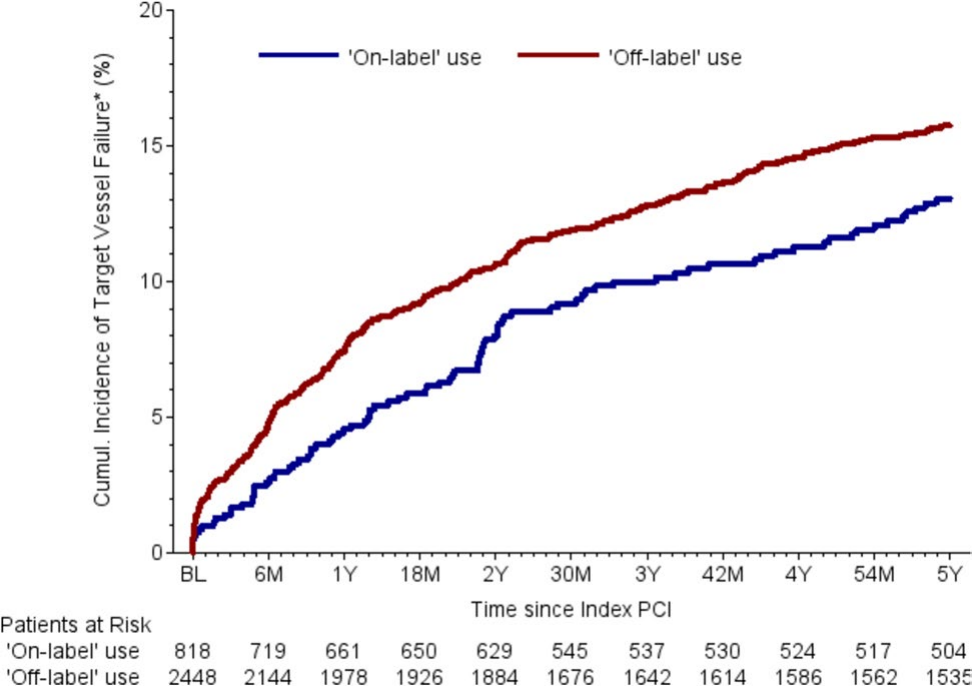
Incidence of target vessel failure

Follow-up data were collected 30 days, 6 months, 2 years, and 5 years after BRS implantation. The list of relevant parameters and the differences between two groups can be found in Table 5. Relevant differences were found in the rates of stent thrombosis after 30 days and target vessel failure (TVF) after 6 months after BRS implantation. Patients with off-label indications had statistically significant higher rates of stent thrombosis after 30 days (1.08% vs. 0.26%, *p* = 0.04) and TVF after 6 months (4.62% vs. 2.61%, *p* = 0.02) (Fig. 1). There are no relevant differences between the two groups regarding overall mortality and cardiovascular mortality (Fig. 2**)**. Numerical differences between the two groups that just failed to reach statistical significance were observed for TVF (17.23% vs. 14.66%, *p* = 0.14) and MACE rates after 5 years (20.11% vs. 16.99%, *p* = 0.09) (Table 5 and Figs. 3–4).

**Table 5:** Follow-up data

	Total	Off-label	On-label	*p*-value*	OR (95% CI)
30-day follow-up data					
Patients with 30 d FU record	96.1%	96.1%	96.2%	0.87	0.96 (0.63–1.48)
Alive at follow-up	99.6%	99.6%	99.5%	0.50	1.52 (0.45–5.05)
Confirmed cardiovascular death	0.32%	0.30%	0.39%	0.70	0.77 (0.20–2.98)
Confirmed non-cardiovascular death	0.03%	0.04%	0.00%	0.57	-
BRS thrombosis					
ARC definite	0.88%	1.08%	0.26%	0.04	4.16 (0.98–17.61)
- Probable	0.32%	0.26%	0.52%	0.27	0.49 (0.14–1.75)
- Definite or probable	1.17%	1.29%	0.78%	0.25	1.66 (0.69–4.00)
TLF	1.49%	1.64%	1.05%	0.24	1.58 (0.73–3.40)
TVF	1.72%	1.94%	1.05%	0.10	1.87 (0.88–3.99)
MACE	1.98%	2.16%	1.44%	0.22	1.51 (0.78–2.92)
6-month follow-up data					
Patients with 6 m FU record	96.9%	96.9%	96.7%	0.83	1.05 (0.66–1.67)
Alive at follow-up	99.5%	99.5%	99.5%	0.98	1.01 (0.33–3.15)
Confirmed cardiovascular death	1.27%	1.25%	1.31%	0.91	0.96 (0.46–1.97)
Confirmed non-cardiovascular death	0.23%	0.17%	0.39%	0.27	0.44 (0.10–1.97)
BRS thrombosis					
ARC definite	1.10%	1.29%	0.52%	0.08	2.50 (0.88–7.11)
- Probable	0.45%	0.43%	0.52%	0.74	0.82 (0.26–2.64)
- Definite or probable	1.52%	1.68%	1.05%	0.21	1.62 (0.75–3.48)
TLF	2.73%	3.02%	1.83%	0.08	1.67 (0.94–2.98)
TVF	4.12%	4.62%	2.61%	0.02	1.80 (1.11–2.93)
MACE	4.57%	4.92%	3.53%	0.11	1.41 (0.92–2.17)
2-year follow-up data					
Patients with 2j FU record	96.5%	96.6%	96.1%	0.51	1.16 (0.75–1.77)
Alive at follow-up	100.0%	100.0%	100.0%	-	-
Confirmed cardiovascular death	3.11%	3.02%	3.40%	0.60	0.89 (0.56–1.40)
Confirmed non-cardiovascular death	0.75%	0.69%	0.92%	0.53	0.75 (0.31–1.84)
BRS thrombosis					
ARC definite	1.92%	2.16%	1.17%	0.10	1.87 (0.88–3.98)
- Probable	0.71%	0.66%	0.88%	0.56	0.75 (0.29–1.96)
- Definite or probable	2.59%	2.77%	2.04%	0.29	1.37 (0.76–2.47)
TLF	7.09%	7.36%	6.25%	0.32	1.19 (0.84–1.69)
TVF	10.27%	10.87%	8.43%	0.07	1.32 (0.98–1.79)
MACE	11.68%	12.18%	10.14%	0.15	1.23 (0.93–1.62)
5-year follow-up data					
Patients with 5j FU record	96.6%	96.7%	96.3%	0.66	1.11 (0.71–1.72)
Alive at follow-up	95.9%	95.7%	96.5%	0.37	0.82 (0.53–1.27)
Confirmed cardiovascular death	7.03%	7.10%	6.82%	0.79	1.04 (0.76–1.44)
Confirmed non-cardiovascular death	1.79%	1.69%	2.10%	0.46	0.80 (0.45–1.44)
BRS thrombosis					
ARC definite	2.72%	2.74%	2.66%	0.92	1.03 (0.58–1.82)
- Probable	1.05%	1.06%	1.00%	0.90	1.06 (0.42–2.65)
- Definite or probable	3.71%	3.73%	3.63%	0.91	1.03 (0.63–1.67)
TLF	12.27%	12.59%	11.26%	0.38	1.14 (0.85–1.51)
TVF	16.61%	17.23%	14.66%	0.14	1.21 (0.94–1.56)
MACE	19.36%	20.11%	16.99%	0.09	1.23 (0.97–1.56)

Values are mean ± standard deviation (SD) or number and percentage (*n*, %)

*ARC* academic research consortium, *BRS* bioresorbable scaffold, *FU* follow-up, *MACE* major adverse cardiac events, *PCI* percutaneous coronary intervention, *TLF* target lesion failure, *TVF* target vessel failure

^*^Comparison between off-label and on-label use. The *p*-values are from chi-squared test or Mann–Whitney–Wilcoxon test

## Discussion

The development of BRS was intended to revolutionize interventional cardiology through the potential benefits of bioresorbable materials. Unfortunately, Absorb BRS did not fulfill all the expectations and was withdrawn from the market due to its inferiority to DES. The reasons for this were increased rates of stent thrombosis and target lesion revascularization shown in several studies and meta-analysis [12–14].

GABI-R registry provides many insights into the special features of Absorb BRS. A particular advantage of this real-world registry is the observation phase of up to 5 years and a low rate of loss to follow-up very high rate of patients who took part in the follow-up. Malapposition, acute disruption, underemployment, device-vessel mismatch, incomplete coverage of lesion (geographical miss), overlap, and late discontinuity are the most frequent mechanisms underlying acute and subacute stent thrombosis after implantation [14–16]. A higher rate of improved implantation techniques and the use of intravascular imaging could certainly reduce the rate of subacute stent thrombosis, even in off-label indications. Although there was a general recommendation for post-dilation after BRS implantation and the use of PSP criteria, this was not followed by all operators. The same applies to the use of intracoronary imaging, which was used relatively rarely. These aspects, which at first glance seem negative, reflect real-life conditions in comparison to approval studies [17, 18]. Patients with more complex coronary anatomy have higher rates of stent thrombosis than patients who were treated with simpler lesions. The differences are statistically significant within the first 30 days of observation (1.08% vs. 0.26%, *p* = 0.04). After 5 years, however, the cumulative stent thrombosis rates are similar between the two groups (2.74% vs. 2.66%, *p* = 0.92).. Nakamura et al. sheds light on the long-term follow-up data of 135 patients from Japan with follow-up data of 5 years [19]. Special features of the study are that the selected patient population is small, but the BRS implantation technique is rather optimal with a proportion of intracoronary imaging of 91.4% and the use of the PSP technique in almost all patients (98.6%). The selection criteria for patients and the treated lesions comply with the on-label criteria of our study. No definite/probable stent thrombosis was reported through 5 years, and the cumulative TLF rate was 5.1% (6/118). The results of this study demonstrate that optimal implantation technique reduces the risk of stent thrombosis and device-associated complications through 5 years [19].

A notable feature of the results of this study is that the rate of stent thrombosis in both groups converges after approximately 2 years, with the two subgroups showing similar outcomes within this timeframe. A possible explanation for the increase in BRS thrombosis up to 2 years after implantation is the biodegradation of the scaffold matrix. The thrombogenicity of scaffold matrix components during the biodegradation process of the polymer is higher than that of the polymeric material itself [20]. Why the on-label group in this study experiences such an increase remains unclear. It can be speculated that these cases of stent thrombosis may also be attributed to suboptimal implantation techniques, as heterogeneous endothelialization of the scaffold struts could lead to exposure of biodegradation products into the bloodstream, resulting in a high risk of thrombus formation. This phenomenon might affect the on-label group more frequently because suboptimally implanted BRS in the off-label group tend to develop stent thrombosis much earlier. The phenomenon of an increase in BRS thrombosis between 20 and 24 months after implantation was also observed in a large-scale meta-analysis involving 3884 patients from four ABSORB trials conducted at 301 centers in North America, Europe, and Asia, indicating that this phenomena does not appear to be specific to our registry [21]. After completed biodegradation, approximately 2 years post-implantation, the rates of BRS thrombosis in the two subgroups are equal. Other observational studies also show comparable results in terms of stent thrombosis and TLF rates [21, 22].

Some insights into the development of the scaffold after implantation were provided by OCT-based studies. In a study with Absorb BRS, intravascular imaging using OCT was performed post-procedure and at 6, 12, 24, and 36 months. The rate of acute scaffold disruption was 3.9%, while late strut discontinuity was observed in approximately 40% of patients [23]. The authors attributed acute scaffold disruption to overexpansion of the BRS at the time of implantation. Late strut discontinuity, on the other hand, was described as a normal part of the scaffold’s biodegradation process. Another important aspect is the duration of dual antiplatelet therapy (DAPT) received by BRS patients. For the participants in the registry, therapy was not determined by the registry itself but was left to the discretion of the local operators. In most cases, DAPT for one year was recommended. The proportion of patients still on DAPT 2 years after implantation was 15.4%. This could partly explain cases of late thrombosis, as platelet inhibition may have been insufficient during the biodegradation phase. Based on current knowledge, an extended DAPT regimen of up to 3 years after implantation would have been a safer option. These characteristics of BRS, such as the need for precise lesion preparation and post-treatment, the ideal use of intravascular imaging, and higher rates of stent thrombosis during “complicated” biodegradation, make BRS less attractive compared to conventional DES. A pooled meta-analysis compared over 3000 patients with BRS and everolimus-eluting stents in long-term follow-up (> 5 years) across four studies [21]. The results showed that BRS had higher event rates compared to DES in terms of TLF (14.9% vs. 11.6%, *p* = 0.03) and cumulative stent thrombosis rates (2.5% vs. 0.8%, *p* = 0.02). Very late stent thrombosis, occurring 3–5 years after implantation, was rare in BRS (0.3%) but three times higher compared to DES (0.1%). This difference also indicates that biodegradation is more problematic compared to the epithelialization observed with conventional DES.

The numerical difference in MACE rate (off label group 20.11% vs on label group 16.99, *p* = 0.09) can be explained by the fact that the off-label group generally includes more patients with severe coronary disease, who also suffer more cardiovascular events in the long term. Even though the Arbsorb BRS have not been available on the market since 2017, the results of the long-term studies with an observation period of 5 years are only now being published and have impact on the development of absorbable stent technology. These facts support the postulate that strict adherence of a BRS-specific implantation strategy leads to a significant reduction in device-associated complications. For a while, there was also hope that magnesium-based resorbable scaffold would have these properties and be better able to adapt to the wall. The latest results show that compared to the standard DES, the magnesium BRS unfortunately has lower angiographic efficacy, a higher rate of TLR [24, 25]. Currently, other new generations of magnesium scaffolds are being investigated; the first results are promising [26]. Some devices—such as the Drug-Eluting Resorbable Magnesium Scaffold (DREAMS 2G, Magmaris® Biotronic) and Fantom® (REVA Medical), made from desaminotyrosine polycarbonate—are currently available on the market. The initial clinical results after a 6–12-month observation period, as well as intravascular imaging, are very promising [26, 27]. There are now also 5-year reports available for the Fantom bioresorbable scaffold; however, these are based on a small patient population (240 patients) [28], so it remains unclear whether the bioresorbable approach is superior to conventional DES. The challenge appears to be to find a balance between device flexibility and stability in bioresorbable materials. There are currently no devices available on the market that can optimally combine these qualities and are proved superior to conventional DES [29, 30]. Ideally, future research should also take into account that the majority of patients have more complex coronary lesions and that future BRS should also be suitable for patients with off-label indications.

### Limitations

Since this is a data evaluation from the registry, the following points should be considered as limitations. The decision whether to implant BRS for the off-label or on-label indication was not pre-specified and was left to the operator. The evaluation of the vessel size was not pre-specified, and different modalities (visual sizing or different intravascular imaging) were used. PSP criteria were rare adopted in both groups. Overall, intravascular imaging methods were used in a small proportion of patients. As most patients were included to the registry after BRS implantation, the real-life rate of periprocedural complications can be underestimated.

## Conclusion

The off-label use of BRS is associated with a higher rate of stent thrombosis in the short term and in the long term with higher MACE events considering more complex lesions and a higher morbidity. In the long term, there are no differences regarding stent thrombosis.

## Abbreviations

ACS: Acute coronary syndrome
ARC: Academic research consortium
BRS: Bioresorbable scaffold
CTO: Chronic total occlusion
DAPT: Dual antiplatelet therapy
DES: Drug eluting stent
GABI-R: German-Austrian Absorb Registry
IHF: Institut für Herzinfarkt Forschung
MACE: Major adverse cardiovascular events
MI: Myocardial infarction
PCI: Percutaneous coronary angioplasty
SD: Standard deviation
STEMI: ST-elevation myocardial infarction
TLF: Target lesion failure
TLR: Target lesion revascularization
